# Temporomandibular Joint Anatomy Assessed by CBCT Images

**DOI:** 10.1155/2017/2916953

**Published:** 2017-02-02

**Authors:** Silvia Caruso, Ennio Storti, Alessandro Nota, Shideh Ehsani, Roberto Gatto

**Affiliations:** ^1^Department of Life, Health & Environmental Sciences, University of L'Aquila, L'Aquila, Italy; ^2^Dental School, Vita-Salute San Raffaele University, Milan, Italy; ^3^Department of Dentistry, University of Tor Vergata, Rome, Italy

## Abstract

*Aim*. Since cone beam computed tomography (CBCT) has been used for the study of craniofacial morphology, the attention of orthodontists has also focused on the mandibular condyle. The purpose of this brief review is to summarize the recent 3D CBCT images of mandibular condyle.* Material and Methods*. The eligibility criteria for the studies are (a) studies aimed at evaluating the anatomy of the temporomandibular joint; (b) studies performed with CBCT images; (c) studies on human subjects; (d) studies that were not clinical case-reports and clinical series; (e) studies reporting data on children, adolescents, or young adults (data from individuals with age ≤ 30 years). Sources included PubMed from June 2008 to June 2016.* Results*. 43 full-text articles were initially screened for eligibility. 13 full-text articles were assessed for eligibility. 11 articles were finally included in qualitative synthesis. The main topics treated in the studies are the volume and surface of the mandibular condyle, the bone changes on cortical surface, the facial asymmetry, and the optimum position of the condyle in the glenoid fossa.* Conclusion*. Additional studies will be necessary in the future, constructed with longitudinal methodology, especially in growing subjects. The limits of CBCT acquisitions are also highlighted.

## 1. Introduction

Radiological images are indispensable in orthodontic diagnosis, as they have increased the accuracy of diagnosis. Since three-dimensional (3D) diagnostic imaging has been diffused for the study of craniofacial morphology, the attention of orthodontists has also focused on the study of the anatomy of the mandibular condyle. As the condyle is the primary center of growing in the mandible and is a special cartilage (secondary cartilage), it answers the continuous stimuli through a remodeling process from childhood to adulthood [1]. The functional stimuli act through a remodeling of the subchondral bone volume, as assessed in rats and mice [2] (the most prevalent bony changes of the condyle are, for example, the flattening, the erosion, the sclerosis, the presence of osteophytes, and the resorption) [3]. Functional stimuli can be represented also by a disc displacement as shown in the rabbit joints [4] which was demonstrated to cause an enlargement of the condyle, which is in part caused by hyperplasia of the condylar cartilage and partly by an increase of the surface area of nonarticulating portion of the condyle.

The study of the condylar morphology is important to understand the complex mechanism of interdependence between form and function during the growing process.

Cone Beam Computed Tomography (CBCT) has been introduced as CBCT scanner in 1982 at Mayo clinic; it began to be marketed during 1990s [5]. Initially, the CBCT scanners had been extensively used in the field of medical clinical imaging [6]. Since then, the CBCT scanner systems have evolved a lot. Today, they are used in various sectors of diagnostic imaging in medicine [6]. The CBCT images are considered very useful in joint visualization of the temporomandibular joint (TMJ), among all the other methods [7]: (a) the* panoramic radiograph* does not show very well the condylar anatomy, its variation, and its adaption to the functional stimuli; it is a simple exam to obtain; so it is very easy to be obtained but not always useful [8]. (b) The* lateral radiographs* of the skull present too much overlapping images of other anatomical structures; in addition they do not visualize soft tissues. (c) The* correct axial tomography* gives a well view of the erosions and osteophytes on the surface of condyle [9], but their visualization and interpretation are not easy, as the other structures are often not represented to reduce overlap; so it is not easy to study the whole anatomy of the condyle [7]. (d) The* computed tomography* (CT) is valid, in both clinics and research, for the visualization the mandible [10] but the device has a high cost, so it was finally confined in public hospital structures because it requests infrastructure [5].

The CBCT images are considered to evidence very well the temporomandibular joint (TMJ). The purpose of this brief review is to summarize the recent 3D CBCT images of mandibular condyle.

## 2. Material and Methods

The eligibility criteria for the studies are as follows: (a) studies aimed at evaluating the anatomy of the temporomandibular joint; (b) studies performed with CBCT images; (c) studies on human subjects; (d) studies that were not clinical case-reports and clinical series; (e) studies reporting data on children, adolescents, or young adults (data from individuals with age ≤ 30 years). No language restriction was applied. Sources included PubMed from June, 2008 to June, 2016. A search strategy using three different keywords was developed, including “anatomy,” “CBCT,” and “mandibular condyle” (this latter term has also been flanked with “temporomandibular joint,” “glenoid fossa,” “temporomandibular joint disc,” and “TMJ”). The terms were combined together using the Boolean conjunction “and.” Additionally, hand searching was applied to the reference list of retrieved articles.

### 2.1. Data Extraction

Data were extracted from the studies about the sample size, both the number of individuals and the number of TMJs observed, because both the right and left TMJs were observed in the majority of the studies, and the results are reported with particular reference to the differences between left and right TMJs (to investigate the symmetry). The data were extracted relative to the gender distribution and range of age in the samples. Furthermore, the methodological structure of the same research has been extracted for each study. Finally, for each study, data about the results were reported (Table 1). The quality of the studies is established by the assignment of scores to each full-text article included in the qualitative analysis. The quality of each study, with a maximum possible score of 11, was considered as follows: low: total score ≤ 4; medium: 5 ≤ score ≤ 8; high: score ≥ 9 (Table 2).

## 3. Results

**Figure 1:**
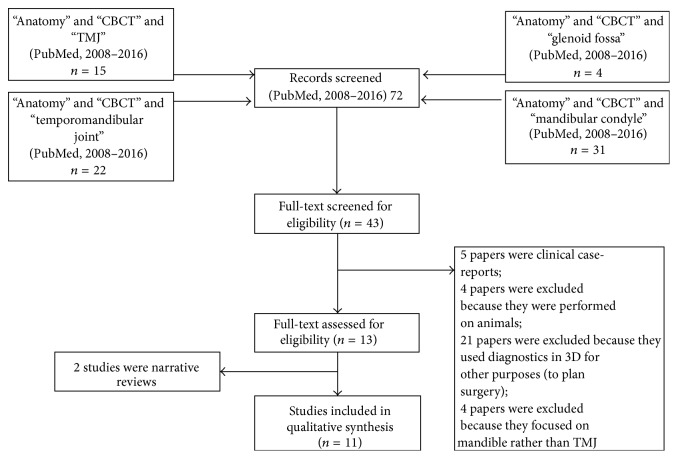
Flow chart of the study.

The results of the searches are summarized in a flow chart of Figure 1.

43 full-text articles were initially screened for eligibility. Then the following papers were excluded: 5 papers were clinical case-reports; 4 papers were excluded because they were performed on animals; 21 papers were excluded because they used diagnostics in 3D for other purposes (to plan surgery); 4 papers were excluded because they focused on mandible rather than TMJ. Finally, 13 full-text articles were assessed for eligibility. Two articles were review, and 11 articles are finally included in the qualitative synthesis. One study is with case-control construction; two studies with cross-sectional structure. All the other studies are observational analyses. More data about the studies are reported in Table 1.

The quality level of the studies was judged to be medium for ten studies and low for one study (Table 2). Thus, the overall level of the studies on this topic is judged to be merely adequate. The level of quality is mainly affected by the lack, actually in literature, of prospective longitudinal reports that could really clarify what happens inside the TMJs during the long period of craniofacial growth and development.

## 4. Discussion

The purpose of this brief review is to summarize data on the study of TMJ through CBCT images during the period of craniofacial growth development (data derived from samples of children, adolescents, and young adults, i.e., individuals aged ≤ 30 years). A great body of literature concerning the TMJ visualization in CBCT images dates back to recent years. Contrary to what one might consider, CBCT images are not widely used to study the pure anatomy of the joints.

The findings of the studies included in this review can be divided into the following broad topics: (a) the evaluation of the volume and surface of the mandibular condyle; (b) the visualization of the bone changes on the cortical surface; (c) the comparison between the two condyles in cases of facial asymmetry; (d) the linear dimensions of the condyle; and (e) the optimum position of the condyle in the glenoid fossa.

### 4.1. CBCT 3D Imaging Allows the Calculation of Volume and Surface of the Mandibular Condyle

CBCT three-dimensional reconstructions allow the calculation of volume and surface of the mandibular condyle. Saccucci et al. (2012) [11] report that, in Caucasian young adults and adolescents (data from a sample of individuals with a range of 15–29 years, mean age 19.2 years), with varied malocclusions, free of pain or dysfunction of TMJs, the condylar volume is 691.26 ± 54.52 mm^3^ in males and 669.65 ± 58.80 mm^3^ in females and significantly higher in the males compared to females. The same is observed for the condylar surface, although without statistical significance (406.02 ± 55.22 mm^2^ in males, and 394.77 ± 60.73 mm^2^ in females). The Morphometric Index (MI, the volume to surface ratio) is 1.72 ± 0.17 with no significant difference between males and females or the right and left sides.

The same authors report that, in Caucasian young adults and adolescents (15–30 years old) [12], subjects in skeletal class III have a significantly higher condylar volume, with respect to class I and class II subjects, while class II subjects show lower condylar volume, with respect to individuals with class I and class III skeletal relationship.

In Caucasian young adults and adolescents [11] mandibular condyle shows a significantly higher volume and surface in low angle subjects (individuals with low mandibular divergence), compared to the high and normal angles groups (individuals with high and normal mandibular divergence).

By the calculation of condylar volume and the index of asymmetry between the right and the left condylar volumes, CBCT three-dimensional reconstructions allow the diagnosis of early stages of juvenile idiopathic arthritis (JIA) in growing individuals (data derived from a sample of 14 girls and 6 boys with a mean age of 11.21 ± 3.54) [13]. Condylar head volume in growing individuals with JIA is about 844 mm^3^ (median value), with a statistically significant asymmetry between the right and the left condyles of about 26% in their volume. In addition, the analysis of CBCT images allows the detection of early* qualitative* signs of JIA that range from small erosions within the cortex to almost complete deformation of the condylar head [13]. It has been stated that early stages of JIA can be detected by CBCT imaging even before this disease has yet caused damages to the individual's facial development during growth [14].

### 4.2. CBCT 3D Imaging Improves Qualitative Analysis of Condylar Surface and Allows Detecting Mandibular Condylar Shape

In Caucasian young adults and adolescents (data from a sample with mean age 19.2 years; range: 15–29; 74 males and 76 females), CBCT three-dimensional reconstructions allowed establishing that the shape of the condylar head is more frequently* round*, compared to* oval*, followed by a* flattened* form, and, at last, a* spiked* form [15]. In addition, it has been established that CBCT images allow well evidence of signs of adaption (*qualitative analyses*) on the condylar surface [16], whatever the age of the individual is (data derived from a sample of 42 TMJs obtained from 21 dry human skulls, not identified by age, sex, or ethnicity and with no demographic data available). Consequently, the qualitative view of the condylar head surface, looking for signs of adaption, is one of the most common clinical applications of the CBCT images, as also pointed out in a recent review of the literature [17]. Degenerative changes of the mandibular condyle are undeniably more common in individuals over 40 years old [3], because the prevalence of bone changes increases with age [17], but about 40% of young individuals aged 10–29 years seem to show bone changes in their TMJs, and these bone changes are well detectable using CBCT images (data extrapolated from 52 individuals ranged 10–29 years old, adolescent and young adult subjects, included in a greater sample, aged between 10 and 89 years) [17]. Among the bone changes, the small* erosions* of the surface are the more common among adolescents and young adults and can be effectively detected with CBCT images using a FOV (field of view) of 6 inch. The* flattening* and the* osteophytes* are instead more frequent in adults and old subjects and well detected by CBCT images [17]. CBCT is additionally intended to be the superior method to acquire adequate information on the extent, the nature, and precise location of TMJ fractures, in growing individuals who have suffered severe maxilla-facial trauma, with the involvement of the TMJ [18]. CBCT images are not suitable to view inflammatory reactions (e.g., marrow oedema) or to view synovia or cartilage or changes in the deeper zones of the condylar head (e.g., cysts), because the segmentation of the structures, based on the thresholding, is restricted to the delineation of the cortical region in those cases where the cortex had not yet reached its final maturity and density, as it happens in growing individuals [13]. Therefore, in these cases, the segmentation is restricted to delineating only the cortical region, without taking possible changes in the deeper zones into account.

### 4.3. CBCT 3D Imaging Improves the Accuracy of Linear Measurements of Mandibular Condyle (Width, Length, and Height)

The most recent literature states that CBCT images are reliable to evaluate the linear measurements of the condyle: the condylar length (linear distance between anterior point of mandibular condyle and posterior point of mandibular condyle), the condylar width (linear distance between the lateral point and the medial point of mandibular condyle), and the condylar height (linear distance between superior point of mandibular condyle and mandibular lingula), as ascertained by data obtained from a sample of 25 dry skulls, for which the age and gender distribution were not known [19]. One of the latest recommendations, on this topic, is that the linear measurements of the condyle appear more accurate when they are measured through images at density levels below those recommended for osseous examination, which extend into the soft tissue range. These lower density levels may compromise the clinician's capacity to view the bone topography of the whole condylar structure. Consequently, when the aim is to perform the linear measurements of the condyle, it will be not appropriate to perform a global analysis on its shape and volume by the same images and vice versa.

### 4.4. CBCT 3D Imaging Clarifies That, in Cases of Facial Asymmetry, Mandibular Condyles Are Often Symmetric, While Joint Space Can Change between the Two Sides

One of the principal clinical situations associated with the study of the condylar morphology is that of facial asymmetry, mostly in groups of children, adolescents, or young adults.

The CBCT images have contributed to clarifying that in spite of the facial asymmetry, the size of the condyles does not seem to differ between the two sides of the face, in young adult subjects, while what seems to suffer from facial asymmetry is mostly the vastness of the intra-articular spaces. In other words, during the craniofacial growth period, it is witnessing a continuous shift of the condyles in the articular fossae, and this may result in differential growth phenomena between the two sides, but it seems that these differences should not necessarily lead to anatomical asymmetry of the mandibular condyles, while what seems to suffer of facial asymmetry is mostly the vastness of the intra-articular spaces. The TMJ intra-articular spaces are well detectable by CBCT images [20], as showed by a small case-control study which recruited 5 patients diagnosed with facial asymmetry (the cases, 3 females and 2 males with mean age: 24.8 ± 2.9 years) and 5 asymptomatic subjects (the controls, 3 females and 2 males, with mean age, 26 ± 1.2 years) [20]. While no difference in the size of condyle (the coronal condylar width) in either group was observed between the two sides or between the study and the control subjects, some differences were detected in joint space. The superior space of the joint became significantly smaller, compared to control individuals, in both nondeviation and deviation side. Because of the small sample (5 cases and 5 controls), however, it should be emphasized that the data from this study have at least a doubtful reliability.

In addition, in young adults with facial asymmetry, the coronal condylar angle appears significantly different between the two sides and also remarkably larger with respect to asymptomatic subjects [20]. In individuals with facial asymmetry the angle measures 19.18 degrees. In addition, the horizontal condylar angle in young adults with facial asymmetry also is significantly larger than in the asymptomatic subjects, no matter on the nondeviation or the deviation side. This increase of the condylar angles may probably cause the rise of the condylar ridge, and then it may lead to the aggravation of the squeezing in the disc and other soft tissues in the TMJ [20]. In individuals with facial asymmetry, the medial and the anterior joint spaces are different between the left and right sides [16], because the spaces of the nondeviation side are significantly smaller than those on the deviation side. Meanwhile, the medial space in the patients group is significantly smaller than that in the control group on both sides. The lateral and the superior joint space of patients with facial asymmetry appear observably larger than for asymptomatic subjects.

The authors indicate that the decrease of the joint space for the patients with facial asymmetry may lead to the articular disc suffering severe squeezing. This severe squeezing may lead to the joint pain, disc perforation, or other TMJ dysfunctions, which are the common symptoms of the TMJ dysfunction. Data detected from children with occlusal asymmetry (from a sample of 20 Brazilian children, aged 7–10 years: 9 males, mean age 7.9, and 11 females, mean age 8.2 years, affected by unilateral posterior cross-bytes, without premature contacts or functional mandibular shift but with transverse maxillary deficiency, i.e., not functional cross-byte, but anatomical!), by CBCT images, allow confirming the absence of differences in condylar size between the crossed and noncrossed sides [21]. In children, mandibular condylar width results in about 14.1 ± 1.78 mm in the crossed side and 14.56 ± 1.79 mm in the control side, while condylar length is 6.58 ± 0.85 mm in the crossed side and 6.63 ± 0.66 mm in the control side [21].

### 4.5. CBCT 3D Imaging Clarifies the Position of the Condyle in the Glenoid Fossa

The condylar position in the glenoid fossa is well defined by CBCT images.

In young adults, (data derived from 24 TMJ from a Japanese sample of 22 asymptomatic patients with optimal joints function, ranged 12–26 years, mean age 18 years, 10 males and 12 females) the following optimal joint spaces are reported [22].

The optimum mean joint spaces in the coronal view, that is, the coronal lateral space, the coronal central space, and the coronal medial space, are, respectively, 1.8 ± 0.4 mm, 2.6 ± 0.4 mm, and 2.3 ± 0.4 mm, in males, and 1.8 ± 0.4 mm, 2.7 ± 0.6 mm, and 2.4 ± 0.7 mm in females, respectively, with no significant sex differences in these measurements. The mean spaces from the axial view, that is, medial axial space and lateral axial space, are 2.1 ± 0.6 mm and 2.2 ± 0.7 mm, in males, respectively, and 2.2 ± 0.6 mm and 2.4 ± 0.6 mm, in females, respectively, with no significant sex differences in these measurements [22].

These data clarify that in young individuals joint space is smaller laterally than centrally or medially in the coronal view. In the axial view, instead, data indicate that the condyle is nearly centred within the fossa, when observed axially, in a normal joint. The ratio among lateral, central, and medial spaces in the coronal view is 1 to 1.5 to 1.3, while the ratio between lateral and medial spaces in the axial view is 58% to 52% [22].

*Summary*. One of the most important benefits achieved through the CBCT images is the ability to calculate volume and surface of the condyle, potentially useful to study the TMJ development during growing period [11, 15].

In addition, CBCT images have helped to clarify that, in cases of facial asymmetry, mandibular condyles are symmetric in volume and size (although the condyle is the main growth center of the mandible), while differences are detectable only in the amplitude of mandibular joint spaces.

In addition, thanks to CBCT images, it has been possible to establish the extent of the optimum joint space on coronal and axial planes in the glenoid fossa in young individuals.

The starting point of all these studies is the condition that the mandibular condyle holds remodeling abilities that persist throughout one's life. It is histologically characterized by the presence of a layer of undifferentiated germinative mesenchyme cells, a layer of cartilage, and the presence of islands of chondrocytes in the subchondral trabecular bone. This adaptable structure allows answering different force vector against the condyle during mastication [23].

Although the CBCT images have clarified form, volume, surface, location, and symmetry of the condyles, there is still a need to develop longitudinal research protocols to clarify the normal growing process of the TMJ. Owing to ethical constraints, it is known that is not possible to schedule successive radiographic procedures over time in health subjects, and for this, longitudinal studies are rendered more difficult, although CBCT provides low radiation dose. The lack, actually in literature, of prospective longitudinal reports depends primarily on ethical and overall health reasons, which preclude the possibility of performing repeated acquisitions on healthy individuals.

The segmentation of the mandibular condyle is based on 2D Digital Imaging and Communications in Medicine (DICOM), created with CT data set. Software as the Mimics™ software 9.0 (Materialise NV Technologielaan, Leuven, Belgium) can be used. Each condyle must be visualized in the recommended bone density range (range of gray scale from 1350 to 1650) and then graphically isolated prior to the 3D measurements. Frankfort horizontal (FH) plane can be constructed by creating a plane from the inferior orbital rim to the superior border of the external auditory meatus. The initial segmentation can be made parallel to the FH plane just above the superior aspect of the condyle [11]. Then, the area of TMJ can be graphically enlarged, and the remaining surrounding structures can be progressively removed using various graphical sculpting tools for the upper, the lower, and the side condylar walls.

The upper limit of the condyle was defined where the first radiopaque area was viewed in the area of synovia; then, for each of the lower sections, the condyle was isolated through the visualization of cortical bone. The lower limit of condyle was traced when the section left the ellipsoidal shape (due to the presence of the anterior crest) and became circular suggesting the level of the condylar neck. Once the computer isolations were made, three-dimensional multiplan reconstructions were produced for each condyle.

Volumetric measurements were made for each condyle with the Mimics automatic function.

Another limitation of the studies available in the current literature is that sometimes, accurately and unequivocally, all the parameters useful to understand how the acquisitions and the image processing have been performed are not indicated. This aspect can affect the reliability about the repeatability of procedures. Moreover, there is still a considerable difficulty by dentists to set and standardize the acquisition parameters of their device, as many studies published in the literature do not report acquisition parameters clearly. In this review, for example, only 3 studies over 11 report accurate and unequivocally complete data about CBCT acquisition. Although, in any case, it can be emphasized that most of the studies report their error analyses method, as observed in 9 out of 11 studies included in this review.

In general, in all the studies, there is a unanimous conclusion in promoting CBCT as a useful aid for displaying the TMJ, although there are still some technical problems, highlighted by several authors.

## 5. Limitations of the CBCT Acquisition

The issue of the artifacts associated with the patient's accidental movements during the acquisition is not yet resolved, which can be a problem in the pediatric population, especially in case of no compliance. In addition, a further technical problem is the Hounsfield Units (HU) distortion, so that CBCT cannot be used to estimate bone density (bone density is estimated using micro-CT). A further limitation is that the decrease of the radiation dose is accompanied by a proportional decrease in image quality, especially with regard to the contrast resolution, so the soft tissues are not displayed well, especially if internally positioned, near to bone structures [24], such as, the TMJ articular disc. Finally, in growing individuals, CBCT imaging has some limitations when it tries to highlight the changes in the deeper structures of the condylar head (e.g., cysts) that are not well detectable with CBCT images if the cortex of condylar head has not yet reached its final maturity and density. This seems to happen because the segmentation of the CBCT images is based on the thresholding and, when the cortex is not mature, is consequently restricted to delineate the cortical region, without taking possible changes in the deeper zones into account.

This brief review represents only an initial classification of recently published data.

Although CBCT helped to clarify some aspects of the morphology of the mandibular condyle (form, volume, surface, location, and symmetry of the condyles) additional studies will be necessary in future, constructed with longitudinal methodology, especially in growing subjects.

## Figures and Tables

**Table 1:** Data from the studies.

Authors	Year	Type of study	Number of TMJs	Sample	Title
Saccucci et al. [11]	2012	Observational study	188 TMJs	94 patients (46 females and 48 males; 15–30 years old)	Resultant rendering reconstructions of the left and right temporal mandibular joints (TMJs) were obtained. Subjects were then classified on the basis of ANB angle in three classes (I, II, III). The data of the different classes were compared.

Saccucci et al. [12]	2012	Observational study	400 TMJs	200 patients (15–30 years old, 95 males and 105 females)	The condylar volume, the area, and the morphological index (MI) were compared among class I, class II, and class III young adult subjects.

Huntjens et al. [13]	2008	Observational study	40 TMJs	20 patients (14 girls and six boys; mean age 11.21 ± 3.54 years)	Condylar asymmetry and a wide variety of condylar destruction patterns were observed in children with juvenile idiopathic arthritis assessed by cone-beam computed tomography.

Zhang et al. [16]	2014	Cross-sectional study	42 TMJs	42 TMJs evaluated by 7 dentists	42 temporomandibular joints were scanned, respectively, with the CBCT units ProMax® 3D (Planmeca Oy, Helsinki, Finland) and DCT PRO (Vatech, Co., Ltd., Yongin-Si, Republic of Korea) at normal and high resolutions. Seven dentists evaluated all the test images.

Barghan et al.	2012	Review	/	/	Application of cone beam computed tomography for assessment of the temporomandibular joints.

Dos Anjos Pontual et al. [17]	2012	Observational study	638 TMJs	319 patients (250 women and 69 men, range 10–89 years old) *Data from adult subjects were excluded*	The differences in percentage of bone changes among the categories of mobility were compared (ipo, iper, normo, and based on mouth opening) and the right and left sides.

Alexiou et al. [3]	2009	Observational study	142 TMJs	71 patients (60 females and 11 males) (20–75 years old) *Data from adult subjects were excluded*	Evaluation of the severity of temporomandibular joint osteoarthritic changes related to age using cone beam computed tomography.

Farronato et al. [14]	2010	Observational study	60 TMJs	30 children (8–13 years old)	The mandible was isolated from other craniofacial structures; the whole mandibular volume and its components' volumes (condyle, ramus, hemibody, and hemisymphysis on right side and on left side) were calculated.

L. Palomo and J.M. Palomo [18]	2009	Review	/	/	Cone beam CT for diagnosis and treatment planning in trauma cases.

Schlueter et al. [19]	2008	Cross-sectional study	50 condyles	/	Three linear three-dimensional measurements were made on each of the 50 condyles at 8 different Hounsfield unit (HU) windows. These measurements were compared with the anatomic truth.

Zhang et al. [20]	2016	Case-control study	20 TMJs	5 patients with facial asymmetry and 5 asymptomatic subjects, mean age, 26 ± 1.2 years	The TMJ spaces and condylar and ramus angles were assessed and compared between the groups.

Illipronti-Filho et al. [21]	2015	Observational study	40 TMJs	9 males (mean 7.9 years) and 11 females (mean 8.2 years)	Dimensional measurements of the condyles between the right and left sides and crossed and noncrossed sides in sagittal and coronal view were made.

Ikeda et al. [22]	2011	Observational study	24 TMJs	10 males, 12 females; range 12–25 years old	Joint-space distances between the condyle and glenoid fossa were measured at the medial, central, and lateral positions in the coronal plane and medial and lateral positions in the axial plane.

**Table 2:** Quality of the studies.

Authors	Type of study	Sample selection adequacy based on age range across the group/s	Sample selection adequacy based on gender across the group/s	Description of at least an error analysis method	Complete description of technical data about CBCT acquisition	Description of blinding procedure	Prior estimation of sample size or a posteriori power analysis	Points
	Longitudinal Construction: 2 Other methods: 1 Not defined: 0	Full: 2 points: properly and clearly detectable data according to age range (i.e., data derived from groups of children or adolescents, or young adults, or adults) Partial: 1 point Not: 0 point	Full: 2 points Balanced distribution of males and females and separate reports for males and female Partial: 1 point No: 0 point	Yes: 1 point No: 0 point	Complete: 2 Partial: 1 Insufficient: 0	Yes: 1 point No: 0 points	Yes: 1 point No: 0 points	

Saccucci et al. [11]	+ Observational study	+ 15–30 years old (adolescents and young adults)	++ 94 subjects: 46 females and 48 males;	+	+	0	+	7

Saccucci et al. [12]	+ Observational study	+ 15–30 years old (adolescents and young adults)	++ 200 subjects: 95 males and 105 females	+	+	0	+	7

Huntjens et al. [14]	+ Observational study of children with Juvenile idiopathic arthritis	+ Mean age 11.21 ± 3.54 (adolescents and children)	+ 20 patients: 6 males and 14 females	0	++	0	0	5

Zhang et al. [16]	+ Cross-sectional study	0 42 TMJs from 21 dry human skulls not identified for age	0 42 TMJs from 21 dry human skulls not identified for gender	+	++	0	0	4

Dos Anjos Pontual et al. [17]	+ Observational study	++ Range 10–89 years old (adolescents, young adults, and adults)	+ 319 subjects: 69 males and 250 females	+	+	0	0	5

Alexiou et al. [3]	+ Observational study	++ Range 20–75 years old (adolescents, young adults, and adults)	+ 71 subjects: 11 males and 60 females	+	+	0	0	6

Farronato et al. [14]	+ Observational study of children with Juvenile idiopathic arthritis	++ 30 subjects range 8–13 years old	+	+	+	0	+	7

Schlueter et al. [19]	+ Cross-sectional study	0 50 TMJs from 25 dry human skulls not identified for age	0 50 TMJs from 25 dry human skulls not identified for gender	0	++	0	0	3

Zhang et al. [20]	+ Case-control study	++ 10 subjects (5 cases and 5 controls) mean age, 24.8 ± 2.9 among cases and 26 ± 1.2 years among controls (young adults)	++ 10 subjects (2 males and 3 females as cases and 2 males and 3 females as controls)	+	+	0	0	7

Illipronti-Filho et al. [21]	+ Observational study	++ 9 males (mean 7.9 years) and 11 females (mean 8.2 years) (children)	++ 20 subjects: 9 males and 11 females	+	+	0	+	8

Ikeda et al. [22]	+ Observational study	+ 10 males and 12 females range 12–26 years old (adolescents and young adults)	++ 22 subjects: 10 males and 12 females	+	+	0	0	6
